# Reference Gene Selection for qPCR Normalization of *Kosteletzkya virginica* under Salt Stress

**DOI:** 10.1155/2015/823806

**Published:** 2015-10-25

**Authors:** Xiaoli Tang, Hongyan Wang, Chuyang Shao, Hongbo Shao

**Affiliations:** ^1^Key Laboratory of Coastal Biology & Bioresources Utilization, Yantai Institute of Coastal Zone Research (YIC), Chinese Academy of Sciences (CAS), Yantai 264003, China; ^2^University of Chinese Academy of Sciences, Beijing 100039, China; ^3^Yantai Academy of China Agricultural University, Yantai 264670, China; ^4^College of Life Sciences, Shandong Agricultural University, Taian 271018, China; ^5^Institute of Agro-Biotechnology, Jiangsu Academy of Agricultural Sciences, Nanjing 210014, China

## Abstract

*Kosteletzkya virginica* (L.) is a newly introduced perennial halophytic plant. Presently, reverse transcription quantitative real-time PCR (qPCR) is regarded as the best choice for analyzing gene expression and its accuracy mainly depends on the reference genes which are used for gene expression normalization. In this study, we employed qPCR to select the most stable reference gene in *K. virginica* which showed stable expression profiles under our experimental conditions. The candidate reference genes were 18S ribosomal RNA (*18SrRNA*), *β*-actin (*ACT*), *α*-tubulin (*TUA*), and elongation factor (*EF*). We tracked the gene expression profiles of the candidate genes and analyzed their stabilities through BestKeeper, geNorm, and NormFinder software programs. The results of the three programs were identical and *18SrRNA* was assessed to be the most stable reference gene in this study. However, *TUA* was identified to be the most unstable. Our study proved again that the traditional reference genes indeed displayed a certain degree of variations under given experimental conditions. Importantly, our research also provides guidance for selecting most suitable reference genes and lays the foundation for further studies in *K. virginica*.

## 1. Introduction

Increasing amount of attention is paid to transcriptome analysis. Actually, transcriptome analysis refers to the identification and measurement of the differentially expressed transcripts. Thus, the key of the transcriptome analysis still stays in the detection of the gene expression profiles. Northern blotting, semiquantitative reverse transcription-PCR, and reverse transcription quantitative real-time PCR (qPCR) are the three most common and frequently used methods [1]. It is due to its high specificity, sensitivity, and extensive quantification range that qPCR has become the first choice for gene expression profiles analysis [2, 3]. Meanwhile, it is also used for the validation of the high throughput sequencing and microarray results [4]. On the other hand, the results of qPCR can be significantly influenced by a series of factors including the condition of the material, the extraction of the RNA, the operational process, and the synthesis of the cDNA [5]. Hence, the internal reference control which acts as a normalization factor is required to minimize the above disturbances. The ideal internal reference genes were supposed to be equally expressed in different samples, developmental stages, and tissues. Only in this way they can be applied to measure the expressions of the other genes [1]. Therefore, the selection of the reference genes is of paramount importance for the veracity of qPCR.

Generally, the reference genes such as 18S ribosomal RNA (*18SrRNA*), *β*-actin (*ACT*), *α*-tubulin (*TUA*), and elongation factor (*EF*) were used for normalization. Their expression levels stay stable under various experimental conditions usually [6]. However, latest studies have shown that no-one reference gene is able to stand stable under different experimental conditions, or in other words we have to select a suitable reference gene for a given situation [7]. Different software tools or statistical procedures have been developed to identify the suitable reference genes for a given experimental condition. For example, the most widely used software tools are BestKeeper, geNorm, and NormFinder. Now a growing number of reports suggest that a specific experiment model needs a corresponding suitable reference gene. With the help of the above software tools, the identification of the reference genes for plants has advanced greatly. Up to now,* Arabidopsis thaliana* [8], wheat [9], barley [10], rice [11], soybean [12], potato [13], grape [14], poplar [15], tomato [16], chicory [17], tobacco [18], longan [19], sugarcane [20],* Brassica juncea* [21], buckwheat [22], tung tree [23], and coffee [23] have been reported about the selection of the appropriate reference genes under various conditions. However, there is not any report about the identification of reference genes so far in* K. virginica* [24].

*K. virginica*, which is also known as seashore mallow (SM), is a perennial halophytic species native to Mid-Atlantic coasts and Southeastern of the United States [25, 26]. It is a noninvasive species newly introduced into China as an important salt-resistant oil crop in 1992. Its stem and tuberous root are suitable material for producing bioenergy [27]. Its salt-resistance ability is strong; for example, it was reported that it could lead to a normal growth and development in adverse environment with 0.3% to 2.5% sodium salt [28]. Therefore,* K. virginica* is considered to be an ideal plant for the investigation of salt-resistance mechanisms. Indeed, many scientists have focused on this characteristic and made some findings [29]. Most of the studies on* K. virginica* only came down to the physiological features including plant growth, water status, potassium concentrations, lipid peroxidation, and soluble sugar contents, yet studies on gene expression and molecular level were rare. On account of that* K. virginica* is a nonmodal plant with little information on gene sequences; thus, the researches on cellular and molecular levels become much harder.

In order to guarantee the accuracy of the qPCR, the selection and determination of the reference gene are of utmost importance. Here we adopted homology-based cloning strategy to acquire the partial sequences of the typical reference genes. Two treatment groups with different time and concentrations salt treatments were used to identify the stable reference genes for verification. The experimental samples comprehensively stand for the salt treatments and the application of the three software tools ensure the accuracy of the statistical analysis. Our results revealed that the commonly used reference genes indeed displayed a certain degree of fluctuations and* 18SrRNA *or the* 18SrRNA* and* ACT* pair will be the wise choice for the gene expression normalization for* K. virginica* under salt treatments.

## 2. Material and Methods

*Statement.* The Yellow River Delta Reserve permitted the collecting of plant samples. The field studies did not involve endangered or protected species. The field also belongs to our institution: The Seaside Wetland Eco-Experimental Station of Chinese Academy of Sciences, Yantai Institute of Coastal Zone Research (YIC), Chinese Academy of Sciences (CAS), Yantai 264003, China.

### 2.1. Plant Sample Preparation

In our study the* K. virginica* seeds were harvested from* The Seaside Wetland Eco-Experimental Station of Chinese Academy of Sciences,* Yellow River Delta, Shandong Province, China. The stripped seeds were sterilized firstly in 70% alcohol for 5 min and 0.1% mercuric chloride for 10 min and then rinsed with sterile distilled water for several times. The sterilized seeds were fostered in liquid MS for germination with a culture temperature at 25°C. The germinal seeds were transferred to plastic pots filled with vermiculite for further cultivation. The condition of culture was kept at 16 h light/8 h dark with 25/18°C with artificial climatic chambers (Huier, China) and the humidity was kept at 65%. The homogeneous two-week-old* K. virginica* seedlings were used for NaCl treatments. The seedlings in three plastic pots (about 15 plants) were used for an experimental sample. The harvested samples (whole plant) were quickly frozen in liquid nitrogen and stored in −80°C. For different NaCl treatments over times, the concentration of the NaCl was 300 mM and the processing times were 2 h, 6 h, 12 h, and 24 h, respectively. The samples were harvested at 2 h, 6 h, 12 h, and 24 h after NaCl treatment. For different concentrations, the samples were treated with 100, 200, 300, and 400 mM NaCl for 24 h, respectively.

### 2.2. Total RNA Isolation and cDNA Synthesis

All samples were collected from the corresponding* K. virginica* whole seedlings. Total RNA samples were extracted from young seedlings with Trizol Reagent (Invitrogen Carlsbad, CA, USA). The nucleic acid concentrations and the quality of the RNA were determined by microspectrophotometer NanoDrop 2000C (Thermo Scientific). All RNA samples had a 260/280 ratio at 1.8–2.0 and the ratio of 260/230 >2.0. The synthesis of cDNA was carried out with TransScrip One-Step gDNA Removal and cDNA Synthesis SuperMix (Transgen Biotech). Both Oligo (dT) 18 and random primers were used for the reverse transcription. The 20 *μ*L reaction system was performed at 42°C 30 min and 85°C 5 min. The concentrations of the synthesized cDNA were also measured by microspectrophotometer and then diluted down to 100 ng/*μ*L, which was required for qPCR.

### 2.3. Selection of Candidate Reference Genes and Primer Design

**Figure 1:**
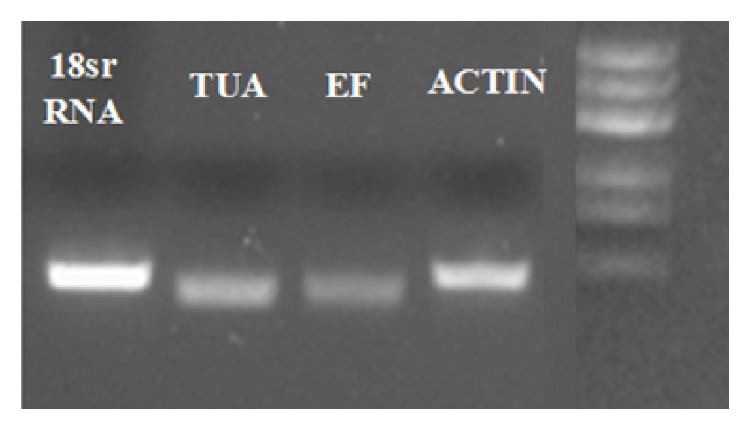
Identification of primer specificity for qPCR amplification by PCR. All detected cDNA were mixed to act as template and the equal amounts of template were used for PCR amplification. 1.0% agarose gel electrophoresis displayed the PCR products of each primer pair.

The most common reference genes in other plants were selected:* ACT*,* EF*,* TUA,* and* 18SrRNA* (Table 1). In view of that the gene sequence of* K. virginica* is almost blank, so the gene sequences of other close relative species are used.* Gossypium hirsutum* is the closest species to* K. virginica* with known genome sequences and most of the reference genes were conserved housekeeping genes. Thus, the sequences of* ACT, EF, TUA, *and* 18SrRNA* in* Gossypium hirsutum* were used to index the conserved and homologous sequence of these genes from the National Center for Biotechnology Information (NCBI) (http://www.ncbi.nlm.nih.gov/) database [30]. The primers for PCR were designed based on those conserved sequences by Primer Premier 5.0. The partial conserved sequences of the candidate genes obtained were at about 600 bp in* K. virginica*. The PCR primers were shown in Table 1. The PCR products were detected by 1.0% agarose gel and displayed expected size and the segment of products were sequenced from Applied Biosystems Invitrogen. The nucleic acid sequences of PCR products were confirmed with BLAST in the National Center for Biotechnology Information too (http://www.ncbi.nlm.nih.gov/). The primers of the candidate reference genes for qPCR were designed by Beacon Designer 7 according to the sequencing results of the PCR products. The qPCR primer sequences were displayed in Table 2. In addition, the qPCR primers amplification specificity of the newly sequenced reference genes was confirmed firstly through RT-PCR with a single product, respectively (Figure 1).

### 2.4. Two-Step Quantitative Real-Time RT-PCR

We carried out qPCR with ABI Prism 7500 FAST (Applied Biosystems, Foster City, CA) and SYBR Green Real-Time Selected Master Mix (Applied Biosystems by Life Technologies) according to the user guide. The reaction volume was 20 *μ*L with 2 *μ*L diluted cDNA, 10 *μ*L 2 × SYBR Master Mix, and 200 nM of each primer. The thermocycling reaction processes were as follows: initial denaturation at 95°C 2 min, 45 cycles of 15 s at 95°C for denaturation of template, and 1 min at 60°C for annealing and extension. The fluorescent dye SYBR Green which was widely used in qPCR can combine with the ds-cDNA to indicate accurately the synthetic cDNA in the reaction system. The fluorescence signal detection was carried out at a temperature of 60–90°C. The primer specificity was confirmed again by the typical melting curve and amplification plot. The Cq values of all the samples were controlled in appropriate scope [31]. All samples were amplified in triplicates and three biological replicates were performed. The Cq values and the corresponding numerical value were imported into Microsoft Excel and used for further analysis.

### 2.5. Data Analysis

The obtained data were converted into the required format according to the different demands of the software tools. Three different applets were used for the data analysis: geNorm (version 3.4), NormFinder (version 0.953), and BestKeeper (version 1.0). The concrete data analysis strategies were described in results. In addition, ANOVA was applied to determine whether the Cq values among the different treatments were significant.

## 3. Results

### 3.1. Selection of Candidate Reference Genes

Due to bottleneck of the extremely limited sequence information of* K. virginica* (L.), there is not any existing gene information which we can adopt directly. By means of homology-based cloning, we at last acquired four genes' fragment (*ACT*,* EF*,* 18SrRNA,* and* TUA*) and designed the qPCR primers based on the obtained partial gene sequences. The selected reference genes for this study displayed different important functions and components in cell. Generally, they were highly conserved and used for normalization of qPCR in many other species. Therefore, we turned to the existing sequence information in GeneBank for PCR primer design. First of all, we obtained a single 600–800 bp PCR product through each pair primer with the expected size, and then the products were sequenced. Thus, we acquired the specific fragment sequence of the candidate genes and in addition the Blastn results showed that these sequences of genes from* K. virginica *were highly homologous with* Gossypium hirsutum*,* Populus trichocarpa, *and* Theobroma cacao* L.  Table 3 shows the comparison results of the obtained sequences in* K. virginica* with* Gossypium hirsutum* and the species with the highest homology. Finally, the primers for qPCR were designed according to the sequencing results and the PCR results showed that all the qPCR primers exhibited a high degree of specificity (Figure 1).

### 3.2. Expression Profiles of the Candidate Reference Genes under Various Salt Treatments in* K. virginica*

**Figure 2:**
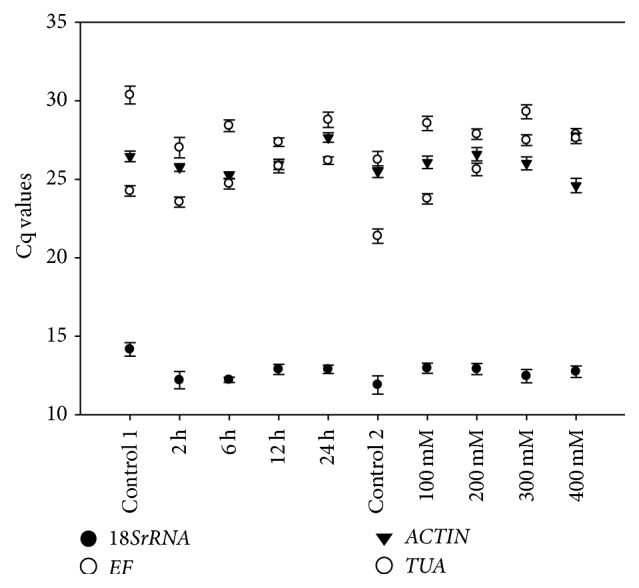
Cq values for the candidate reference genes. The Cq values were used to display the expression profiles of the candidate genes in 10 samples. Control 1 was the sample under normal condition without NaCl treatment in different time period group; control 2 was the sample under normal condition without NaCl in different concentration group.

To assess the expression stabilities of the four candidate reference genes, their expression variations were estimated by qPCR in 10 cDNA samples. The 10 samples belong to two experimental groups. The time group contains 5 samples, namely, control 1, 2 h, 6 h, 12 h, and 24 h. The concentration group includes control 2, 100 mM, 200 mM, 300 mM, and 400 mM NaCl treatments. The qPCR was performed according to the two-step quantitative real-time PCR. All the related parameters of qPCR were controlled in the demanded ranges. The expression profiles of the candidate reference genes were shown in Figure 2. The expression of the candidate genes throughout all samples represented by cycle threshold (Cq) values showed changes to some degree [32]. The Cq values of the studied reference genes fluctuated from 11.71 to 31.14 in all samples of the two experimental groups (Figure 2).

### 3.3. Expression Stability Analysis

Due to the various behaviors of the candidate reference genes, we need some methods of data handling to evaluate the stability of the candidate genes under a certain condition. In order to pick out the optimal reference gene, we adopted the analysis software tools of BestKeeper, geNorm, and NormFinder, which were used extensively in the identification of the reference genes. BestKeeper is an Excel-based tool and it ranks the candidate reference genes with the calculation of the BestKeeper index [1]. Under BestKeeper analysis, the best reference gene shows the strongest correlation with the BestKeeper index. The geNorm is a Visual Basic application tool and gene expression value is evaluated by *M* value. This algorithm compares the *M* values of the candidate genes and eliminates the gene with the highest *M* value, and two genes are left at last. Thus, the last two genes which have the lowest *M* value are regarded as the best pair of the candidate genes [33]. NormFinder is also a Visual Basic application applet. It identifies the optimal reference gene through a model-based approach [34]. Similar to geNorm, a low SD value means a more stable expression profile of the gene in this algorithm.

#### 3.3.1. geNorm

**Figure 3:**
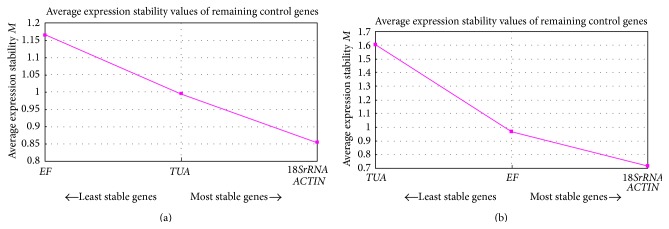
Average expression stability (*M*) values of the candidate genes. The average expression stability (*M*) values are acquired through the stepwise exclusion of the least stable reference gene. Starting from the least stable gene at the left, the genes are ranked according to the ascending expression stability, ending with the two most stable genes at the right. The result of time treatment is shown in (a) and the concentration treatment is displayed in (b).

This software is one of the Visual Basic application tools for Microsoft Excel. It picks out the most stable reference genes from a given sample and figures out the gene expression normalization factors according to the geometric mean of the candidate genes. The parameter employed by geNorm to measure the stability of the candidate gene is the average expression stability (*M*) value. The *M* value is calculated according to the average pairwise variation between all detected genes. The lower *M* value stands for the higher stability of the gene expression [31]. The analysis result of geNorm was displayed in Figures 3(a) and 3(b). For the treatment samples at different points in time,* 18SrRNA* and* ACT* were the most stable reference genes (Figure 3(a)). The* EF* gene was estimated to be the least stable among them. For the samples with different concentration treatments,* 18SrRNA* and* ACT* were the most stable reference genes (Figure 3(b)), while the least stable gene was* TUA*. In addition, the comparison between two experimental conditions suggested that the variations were more significant under different concentrations. Therefore, according to the analysis of geNorm,* 18SrRNA* and* ACT* are the optional reference genes for the normalization of gene expression under NaCl treatments in* K. virginica*.

#### 3.3.2. NormFinder

For further verification, NormFinder was also adopted for the assessment. NormFinder is also a Visual Basic application tool for Microsoft Excel. It is an Add-In for Microsoft Excel; namely, the NormFinder function is added directly to the Microsoft Excel software package. For this algorithm, the more stable genes have lower stability values [34]. NormFinder is able to estimate the intra- and intergroup variations as well as all samples. The assessment results of NormFinder were shown in Table 4. It is worth noting that there were certain differences in the analysis between no subgroups and two subgroups in the stability values. The trend of the two assessments was the same and the no subgroups analysis showed higher stability values. The ranking of the reference genes in terms of their expression stabilities is identical (Table 4).* 18SrRNA* was estimated to be the most stable reference gene in NormFinder with the stability value at 0.064. However,* TUA* showed the most unstable expression profiles with the largest stability value at 0.286 in two subgroups' result. Therefore, the ranking of the candidate reference genes under various NaCl treatments was* 18SrRNA*>*EF*>*ACT*>*TUA*. For no subgroups analysis, the outcome is the same except for the different stability values. Combining the results of geNorm and NormFinder, we can come to a conclusion that the* 18SrRNA* or* 18SrRNA* and* ACTIN *pair should be the best reference genes for gene expression normalization in* K. virginica* under salt treatments.

#### 3.3.3. BestKeeper

BestKeeper is an Excel-based spreadsheet software application. Different from the above two tools, the raw Cq values without any conversion can be loaded in for analysis in BestKeeper. When the original Cq values were imported, the descriptive statistics of each candidate gene were computed, including GM (geometric mean), AM (arithmetic mean), SD (standard deviation), CV (coefficient of variance), Min (minimal), and Max (maximal) [8]. The expression stabilities of the candidate genes were calculated in accordance with the inspection of calculated variations (SD and CV values). The analysis results in this study were displayed in Tables 5(a) and 5(b) and the analysis was also carried out in two ways with all samples and with two subgroups. The calculated results either in all samples or in two subgroups were unanimous with the same two optimal reference genes* 18SrRNA* and* ACT*, yet the least stable reference genes were* EF* and* TUA*. The relative higher Pearson's coefficients of correlation demonstrated that the expression profiles of these genes were altered under NaCl treatment. In brief, the BestKeeper result stayed the same with the outcomes of NormFinder and geNorm. Therefore,* 18SrRNA* or* 18SrRNA* and* ACTIN *pair should be the ideal reference genes for gene expression normalization in* K. virginica* under various NaCl treatments among the four candidate reference genes.

## 4. Discussions

*K. virginica,* a noninvasive species, was imported into China for its salt resistance [33]. Its importance appears due to its ability to survive and develop under high salt environment, which is the goal of salt-resistant crops research. Ever since its introduction, the researches focusing on it are ongoing, but most of them are about physiological researches [1, 29]. The studies at the level of cells and genes are rare. The only reports so far on gene expression detection in* K. virginica* took* ACT* as reference gene for gene expression normalization [31]. Therefore, in order to understand* K. virginica* at the molecular level to find its specific mechanisms for salt-resistance, the selection of the stable reference genes for gene expression normalization in* K. virginica* is imperative. In this study,* 18SrRNA* was demonstrated to be the optimal under salt stress conditions.

The traditional reference genes such as* ACT*,* TUA*, UBQ, and* EF* which are often used as internal reference genes in* Arabidopsis *were also found to alter under some condition [35]. Thus, the selection of the reference genes under a given condition is necessary. Fortunately, we can simplify the complicated confirmation of the reference genes with the help of the designed statistical algorithms. BestKeeper [36], geNorm [33], deltaCq [34], RefFinder [37], and NormFinder [38] are the commonly used software tools to assess the expression stability of the candidate reference genes. In our study, the BestKeeper, geNorm, and NormFinder were employed to calculate the stabilities of the four candidate reference genes in* K. virginica*. The results of the three algorithms revealed that* 18SrRNA* and* ACT* were estimated to be the most stable reference genes in two experiment sets on the basis of the geNorm analysis, while the most unstable genes were* EF* and* TUA,* respectively (Figure 3). Duo to the stepwise exclusion method used by geNorm for stability analysis, there will be two ideal reference genes in Figure 3. As a matter of fact, the *M* values of* 18SrRNA* were smaller than* ACT* in both experimental conditions. For NormFinder analysis, the* 18SrRNA* obtained the smallest stability value with 0.064 which was the same with geNorm outcome (Table 4). The values of the intragroup variation and the intergroup variation of* 18SrRNA* were the minimum among all the candidate genes demonstrating again that* 18SrRNA* was the best choice for this study. But the difference was the second stable reference genes and they were* ACT* and* EF* in geNorm and NormFinder, respectively. The* TUA* was determined to be the worst one again. In BestKeeper, the* 18SrRNA* was still assessed to be the most stable reference gene with the smallest SD value keeping consistent with the previous two assessments. The second stable reference gene was* ACT* with the SD value at 0.61, but its coefficient of correlation (*r*) and *P* value were not ideal.* EF* and* TUA* were estimated to obtain the SD value at 1.00 and 1.51, respectively, which implied that they could not be used for internal reference gene any more. In addition, we can choose more than one reference gene to ensure the accuracy of the study. In this study, the* 18SrRNA* and* ACTIN* pair gave the best performance under salt stress.

Generally,* ACT* is the most widely used internal reference gene under some conditions. Yet, in our research the stability value of* ACT* based on NormFinder analysis was higher than* 18SrRNA *and* EF* (Table 4). Besides,* 18SrRNA* has also been widely used as stable reference gene in many species such as* Arabidopsis* and Rice under particular conditions [39, 40]. Yet Kim et al. found that the expression of* 18SrRNA* can be effected by some biological factor and drug; thus,* 18SrRNA* may not be suitable for biotic stresses [41]. In our study the* 18SrRNA* was proved to be the best reference gene for gene expression normalization in* K. virginica*. Meanwhile,* EF* is another important and widely used reference gene. In the selection of reference gene for Chinese cabbage (*Brassica rapa* L. ssp.* pekinensis*),* EF* was identified as the best choice for normalization in different tissues [34]. Meanwhile,* EF* was also the most stable gene in potato under salt and cold stresses, while the conclusion was not appropriate for Chinese cabbage under the same condition [39]. In our research for* K. virginica* under salt stress,* EF* was not the optimum, especially in the different time treatments under which* EF* was estimated to be the most unstable reference gene (Figure 3(a)). Despite the fact that* TUA* gene also often appeared in the candidate reference genes, our three analysis results all indicated that the expression profile of* TUA* had significant changes with high *M* value (Figure 3), low stability (Table 4), and high SD value (Tables 5(a) and 5(b)) and was not suitable for gene expression normalization in* K. virginica* under salt stress. Similarly, the researches on faba bean (*Vicia faba* L.) [14], banana [42], tomato [1], and* Salvia miltiorrhiza* [1, 17] all revealed that the* TUA* gene was not appropriate for gene expression normalization. In addition, in the research on tomato, the scientists emphasized that we should avoid choosing* TUA* as reference gene because its behavior was far from accepted in their findings [43].

## 5. Conclusion

In summary, our study indicated that the expression stability varied considerably under the various experimental conditions in* K. virginica*. With the help of the software tools of BestKeeper, geNorm, and NormFinder,* 18SrRNA* was identified to be the most stable reference gene among the four candidate traditional reference genes, yet* TUA* appeared to be the most unsuitable reference gene in our analysis. The stable reference gene selected in this study will be very helpful for revealing the gene expression profiles in* K. virginica* under salt stress promoting the realization of it at cellular and gene level. Our study will lay the foundation for further investigation of the salt-resistant mechanism in halophyte as well.

## Figures and Tables

**Table 1:** Candidate reference genes and the PCR primer sequences for *K. virginica*.

Gene name	Function	Primer	Sequence	Length (bp)
*ACTIN*	Structural constituent of cytoskeleton	act-F	GTTGGGATGGGTCAGA	800
		act-R	CCTTGCTCATACGGTCA	

*EF*1*α*	Elongation factor 1 alpha	EF-F	GGTCATTCAAGTATGCCTGG	740
		EF-R	GAACCCAACATTGTCACCAG	

*TUA*	Cytoskeleton structural protein	TUA-F	GTTTTCAGTGCTGTTGGAGGT	700
		TUA-R	AACGCTGGTTGAGTTGGA	

*18SrRNA*	18S ribosomal RNA	18S-F	GAGTATGGTCGCAAGGCTGAA	640
		18S-R	CCTCTAAATGATAAGGTTCAGTGG	

**Table 2:** Candidate reference genes and the qPCR primer sequences in *K. virginica*.

Gene name	Function	Primer	Sequence	Length (bp)
*ACTIN*	Structural constituent of cytoskeleton	actqPCR-F	TTATGTTGCCCTGGACT	160
		actqPCR-R	CCGCTTCCATCCCTA	

*EF*1*α*	Elongation factor 1 alpha	EFqPCR-F	TCAATGAGCCAAAGAGG	120
		EFqPCR-R	CAACACGACCAACAGGA	

*TUA*	Cytoskeleton structural protein	TUAqPCR-F	TATCTCATCTCTCACAGCCTG	119
		TUAqPCR-R	GGGCATACGAGGAAAGCAT	

*18SrRNA*	18S ribosomal RNA	18SqPCR-F	CCGTTCTTAGTTGGTGGA	170
		18SqPCR-R	AACATCTAAGGGCATCACAG	

**Table 3:** Homologous comparison of the candidate genes between *K. virginica* and other species.

Gene name	Function	Blastn (*E* value)	Identity (%)	The specie with highest homolog
*ACTIN*	Structural constituent of cytoskeleton	0.0	94	*Gossypium hirsutum*
*EF*1*α*	Elongation factor 1 alpha	0.0	93	*Theobroma cacao*
*TUA*	Cytoskeleton structural protein	0.0	93	*Gossypium hirsutum*
*18SrRNA*	18S ribosomal RNA	0.0	99	*Pavonia spinifex voucher*

**Table 4:** Analysis of the candidate reference genes in view of the stability values estimated by NormFinder.

Gene name	Stability value (two subgroups)	Stability value (no subgroups)	Intragroup variation		Intergroup variation	
			1	2	1	2
*18SrRNA*	0.064	0.234	0.071	0.052	0.007	−0.007
*EF*1*α*	0.132	0.583	0.651	0.047	0.059	−0.059
*ACTIN*	0.145	0.596	0.120	0.605	0.069	−0.069
*TUA*	0.286	1.164	0.543	2.196	−0.134	0.134

**(a):** 

Gene name	SD [±CP]			Coefficient of correlation (*r*)			*P* value		
	All samples	1	2	All samples	1	2	All samples	1	2
*18SrRNA*	0.43	0.53	0.33	0.76	0.83	0.75	0.001	0.001	0.001
*EF*1*α*	1.00	1.12	0.94	0.82	0.79	0.85	0.001	0.001	0.001
*ACTIN*	0.61	0.68	0.57	0.52	0.75	0.26	0.004	0.001	0.355
*TUA*	1.51	0.88	2.08	0.72	0.50	0.88	0.001	0.056	0.001

**(b):** 

CV [%CP]			Min [*x*-fold]			Max [*x*-fold]			SD [±*x*-fold]		
All sample	1	2	All sample	1	2	All sample	1	2	All sample	1	2
3.41	4.14	2.63	−2.01	−1.65	−1.84	2.85	2.60	1.36	1.35	1.45	1.26
3.56	3.94	3.37	−5.95	−3.44	−5.15	7.92	6.85	3.93	2.00	2.17	1.92
2.35	3.54	2.20	−3.38	−2.06	−2.89	3.67	3.13	1.96	1.53	1.60	1.48
6.03	2.58	8.28	−12.5	−2.62	−13.3	7.59	3.22	7.16	2.85	1.80	4.24

1 stands for the different time period treatment and 2 stands for the different concentration treatment.

## References

[1] Mallona I., Lischewski S., Weiss J., Hause B., Egea-Cortines M. (2010). Validation of reference genes for quantitative real-time PCR during leaf and flower development in Petunia hybrida. *BMC Plant Biology*.

[2] Mafra V., Kubo K. S., Alves-Ferreira M. (2012). Reference genes for accurate transcript normalization in citrus genotypes under different experimental conditions. *PLoS ONE*.

[3] Wong M. L., Medrano J. F. (2005). Real-time PCR for mRNA quantitation. *BioTechniques*.

[4] Artico S., Nardeli S. M., Brilhante O., Grossi-de-Sa M. F., Alves-Ferreira M. (2010). Identification and evaluation of new reference genes in *Gossypium hirsutum* for accurate normalization of real-time quantitative RT-PCR data. *BMC Plant Biology*.

[5] Die J. V., Román B., Nadal S., González-Verdejo C. I. (2010). Evaluation of candidate reference genes for expression studies in *Pisum sativum* under different experimental conditions. *Planta*.

[6] Tong Z., Gao Z., Wang F., Zhou J., Zhang Z. (2009). Selection of reliable reference genes for gene expression studies in peach using real-time PCR. *BMC Molecular Biology*.

[7] Podevin N., Krauss A., Henry I., Swennen R., Remy S. (2012). Selection and validation of reference genes for quantitative RT-PCR expression studies of the non-model crop Musa. *Molecular Breeding*.

[8] Andersen C. L., Jensen J. L., Ørntoft T. F. (2004). Normalization of real-time quantitative reverse transcription-PCR data: a model-based variance estimation approach to identify genes suited for normalization, applied to bladder and colon cancer data sets. *Cancer Research*.

[9] Czechowski T., Stitt M., Altmann T., Udvardi M. K., Scheible W.-R. (2005). Genome-wide identification and testing of superior reference genes for transcript normalization in arabidopsis. *Plant Physiology*.

[10] Paolacci A. R., Tanzarella O. A., Porceddu E., Ciaffi M. (2009). Identification and validation of reference genes for quantitative RT-PCR normalization in wheat. *BMC Molecular Biology*.

[11] Burton R. A., Shirley N. J., King B. J., Harvey A. J., Fincher G. B. (2004). The CesA gene family of barley. Quantitative analysis of transcripts reveals two groups of co-expressed genes. *Plant Physiology*.

[12] Jain M., Nijhawan A., Tyagi A. K., Khurana J. P. (2006). Validation of housekeeping genes as internal control for studying gene expression in rice by quantitative real-time PCR. *Biochemical and Biophysical Research Communications*.

[13] Jian B., Liu B., Bi Y., Hou W., Wu C., Han T. (2008). Validation of internal control for gene expression study in soybean by quantitative real-time PCR. *BMC Molecular Biology*.

[14] Nicot N., Hausman J.-F., Hoffmann L., Evers D. (2005). Housekeeping gene selection for real-time RT-PCR normalization in potato during biotic and abiotic stress. *Journal of Experimental Botany*.

[15] Reid K. E., Olsson N., Schlosser J., Peng F., Lund S. T. (2006). An optimized grapevine RNA isolation procedure and statistical determination of reference genes for real-time RT-PCR during berry development. *BMC Plant Biology*.

[16] Brunner A. M., Yakovlev I. A., Strauss S. H. (2004). Validating internal controls for quantitative plant gene expression studies. *BMC Plant Biology*.

[17] Expósito-Rodríguez M., Borges A. A., Borges-Pérez A., Pérez J. A. (2008). Selection of internal control genes for quantitative real-time RT-PCR studies during tomato development process. *BMC Plant Biology*.

[18] Maroufi A., Van Bockstaele E., De Loose M. (2010). Validation of reference genes for gene expression analysis in chicory (*Cichorium intybus*) using quantitative real-time PCR. *BMC Molecular Biology*.

[19] Schmidt G. W., Delaney S. K. (2010). Stable internal reference genes for normalization of real-time RT-PCR in tobacco (*Nicotiana tabacum*) during development and abiotic stress. *Molecular Genetics and Genomics*.

[20] Lin Y. L., Lai Z. X. (2010). Reference gene selection for qPCR analysis during somatic embryogenesis in longan tree. *Plant Science*.

[21] Iskandar H. M., Simpson R. S., Casu R. E., Bonnett G. D., Maclean D. J., Manners J. M. (2004). Comparison of reference genes for quantitative real-time polymerase chain reaction analysis of gene expression in sugarcane. *Plant Molecular Biology Reporter*.

[22] Chandna R., Augustine R., Bisht N. C. (2012). Evaluation of candidate reference genes for gene expression normalization in *Brassica juncea* using real time quantitative RT-PCR. *PLoS ONE*.

[23] Demidenko N. V., Logacheva M. D., Penin A. A. (2011). Selection and validation of reference genes for quantitative real-time PCR in buckwheat (*Fagopyrum esculentum*) based on transcriptome sequence data. *PLoS ONE*.

[24] Cruz F., Kalaoun S., Nobile P. (2009). Evaluation of coffee reference genes for relative expression studies by quantitative real-time RT-PCR. *Molecular Breeding*.

[25] Guo Y.-Q., Tian Z.-Y., Qin G.-Y. (2009). Gene expression of halophyte *Kosteletzkya virginica* seedlings under salt stress at early stage. *Genetica*.

[26] Gallagher J. L. (1985). Halophytic crops for cultivation at seawater salinity. *Plant and Soil*.

[27] Ruan C.-J., Li H., Mopper S. (2009). Kosteletzkya virginica displays mixed mating in response to the pollinator environment despite strong inbreeding depression. *Plant Ecology*.

[28] Yan K., Chen P., Shao H., Shao C., Zhao S., Brestic M. (2013). Dissection of photosynthetic electron transport process in sweet sorghum under heat stress. *PLoS ONE*.

[29] Zhou G., Xia Y., Ma B. L., Feng C., Qin P. (2010). Culture of seashore mallow under different salinity levels using plastic nutrient-rich matrices and transplantation. *Agronomy Journal*.

[30] Han R.-M., Lefèvre I., Ruan C.-J., Beukelaers N., Qin P., Lutts S. (2012). Effects of salinity on the response of the wetland halophyte *Kosteletzkya virginica* (L.) Presl. to copper toxicity. *Water, Air, & Soil Pollution*.

[31] Bustin S. A., Benes V., Garson J. A. (2009). The MIQE guidelines: minimum information for publication of quantitative real-time PCR experiments. *Clinical Chemistry*.

[32] Altschul S. F., Gish W., Miller W., Myers E. W., Lipman D. J. (1990). Basic local alignment search tool. *Journal of Molecular Biology*.

[33] Pfaffl M. W., Tichopad A., Prgomet C., Neuvians T. P. (2004). Determination of stable housekeeping genes, differentially regulated target genes and sample integrity: BestKeeper—excel-based tool using pair-wise correlations. *Biotechnology Letters*.

[34] Vandesompele J., de Preter K., Pattyn F. (2002). Accurate normalization of real-time quantitative RT-PCR data by geometric averaging of multiple internal control genes. *Genome Biology*.

[35] Gutierrez L., Mauriat M., Pelloux J., Bellini C., Van Wuytswinkel O. (2008). Towards a systematic validation of references in real-time RT-PCR. *The Plant Cell*.

[36] Lee J. M., Roche J. R., Donaghy D. J., Thrush A., Sathish P. (2010). Validation of reference genes for quantitative RT-PCR studies of gene expression in perennial ryegrass (*Lolium perenne* L.). *BMC Molecular Biology*.

[37] Silver N., Best S., Jiang J., Thein S. L. (2006). Selection of housekeeping genes for gene expression studies in human reticulocytes using real-time PCR. *BMC Molecular Biology*.

[38] Xie F., Xiao P., Chen D., Xu L., Zhang B. (2012). miRDeepFinder: a miRNA analysis tool for deep sequencing of plant small RNAs. *Plant Molecular Biology*.

[39] Qi J., Yu S., Zhang F. (2010). Reference gene selection for real-time quantitative polymerase chain reaction of mRNA transcript levels in Chinese cabbage (*Brassica rapa* L. ssp. *pekinensis*). *Plant Molecular Biology Reporter*.

[40] Klok E. J., Wilson I. W., Wilson D. (2002). Expression profile analysis of the low-oxygen response in arabidopsis root cultures. *The Plant Cell Online*.

[41] Kim B.-R., Nam H.-Y., Kim S.-U., Kim S.-I., Chang Y.-J. (2003). Normalization of reverse transcription quantitative-PCR with housekeeping genes in rice. *Biotechnology Letters*.

[42] Gutierrez N., Giménez M. J., Palomino C., Avila C. M. (2011). Assessment of candidate reference genes for expression studies in *Vicia faba* L. by real-time quantitative PCR. *Molecular Breeding*.

[43] Yang Y., Hou S., Cui G., Chen S., Wei J., Huang L. (2010). Characterization of reference genes for quantitative real-time PCR analysis in various tissues of *Salvia miltiorrhiza*. *Molecular Biology Reports*.

